# Doctors and Municipal Socialism

**Published:** 1902-09-13

**Authors:** 


### Doctors and Municipal Socialism.

Those interesting members of the medical profession who take upon themselves the office of appearing in public as the champions of their brethren, and loudly declaring that this or that "must" be remedied in the course of the next session of Parliament, appear to us to be sadly neglecting a work which seems to lie ready to their hands, the work of pointing out how much might be effected by medical men in their own localities in the direction of controlling the present inordinate desire of municipalities to embark in "great" works of real or supposed public utility at the risk, and almost too surely at the cost, of the ratepayers. There were, we believe, certain parishes or districts in the Southern States of America in which, after the civil war, the burdens upon the land exceeded its actual value, so that no one would admit ownership, and it fell out of use and cultivation. This has not yet happened, so far as we are aware, in any parish in the United Kingdom, but it seems, nevertheless, to be the goal towards which many "progressive" municipalities are tending, and tending at a rate which should certainly be checked by the prudence of the community. That eminent person Sir Niketes Brayley, thunders from the platform concerning the duty of the medical profession to make its voice heard, and its strength felt, in parliamentary elections, or concerning the necessity of its being "represented" in the House of Commons; But he neglects to urge upon his hearers the duty of making their voices heard in the narrower field of local administration, a field in which their influence might often be exerted to great advantage, without even an apparent transgression of the limits of real public interests, and which has, moreover, for many years served to educate those who have worked in it for the discharge of more important functions in "another place." A dozen medical men, in a borough of ten thousand inhabitants, can never expect to exert any appreciable influence upon a parliamentary election, which would turn, as a rule, upon some public question on which they themselves would probably be divided. If by any chance this were not the case, and if parties were very evenly balanced, any attempt on the part of the medical practitioners of a town to throw their whole weight into one scale, in return for a pledge to support something which might be represented as a class interest, would bring down upon them universal obloquy. On questions of local administration, on the other hand, there would be much more prospect of unanimity among men who possessed the combined advantages of common sense and of scientific training, and it might often be in the power of the profession, in any locality, to save their fellow ratepayers from being victimised by the fascination which "big" schemes frequently appear to possess for persons of modest means and limited intelligence. It is suggested for example that the corporation should become gas-makers, or purveyors of electric light, or purveyors of water, or that they should construct tramways which would carry their neighbours at least a few miles on the way towards the nearest watering place or health resort. It is unimportant that none of the town councillors have any knowledge of any of the forms of industry in which it is their intention to embark; they have the ratepayers behind them, and everything is sure to turn out well if only they spend money enough. Their gas shall be supplied at a penny a thousand feet less than has hitherto been charged by the company that they intend to supersede, and who cares what the rates will be? Now it is in such emergencies as these that the doctors of a locality might with great advantage agree among themselves as to the merits or demerits of the course proposed, and might bring facts and arguments to the help of their neighbours. If they are ever to gain political influence, it must be by identifying their interests with those of their fellow townsfolk, and not by any premature insistence upon claims of their own. The last two years have shown by many examples that there is no surer road to the loss of influence by professional politicians than that they should even incur the suspicion of preferring the interests of a party to those of the nation; and for doctors to seek such influence as doctors, with the avowed intention of employing it for the furtherance of merely professional objects, would be to invite not only failure but also derision. Their only road to success would be by first showing themselves good and prudent citizens in relation to questions bearing upon the general welfare, and in precise proportion to their success in doing this would be their ultimate success in bringing about a conviction that, for example, the encouragement of quackery by the State is at once immoral in the rulers, and injurious to the best interests of the ruled.

